# Workflow optimization in acute stroke therapy using a mobile application – a pilot study

**DOI:** 10.1177/17562864251379215

**Published:** 2025-10-18

**Authors:** Frieso Geerd Stevens, Hans Worthmann, Clara Zoe Fricke, Mareike Schulze, Gerrit M. Grosse, Maria M. Gabriel, Jan Beneke, Sybille Schiele, Ramona Schuppner, Karin Weissenborn, Friedrich Götz, Anna-Lena Boeck, Johanna Ernst

**Affiliations:** Department of Neurology, Hannover Medical School, Hannover, Germany; Department of Neurology, Hannover Medical School, Hannover, Germany; MHH Information Technology, Hannover Medical School, Hannover, Germany; Peter L. Reichertz Institute for Medical Informatics, Technical University Braunschweig and Hannover Medical School, Hannover, Germany; Department of Neurology, Hannover Medical School, Hannover, Germany; Department of Neurology and Stroke Center, University of Basel, Basel, Switzerland; Department of Neurology, Hannover Medical School, Hannover, Germany; MHH Information Technology, Hannover Medical School, Hannover, Germany; Service Development, Hannover Medical School, Hannover, Germany; Department of Neurology, Hannover Medical School, Hannover, Germany; Department of Neurology, Hannover Medical School, Hannover, Germany; Institute for Diagnostic and Interventional Neuroradiology, Hannover Medical School, Hannover, Germany; Department of Neurology, Hannover Medical School, Hannover, Germany; Department of Neurology, Hannover Medical School, Carl-Neuberg-Str. 1, Hannover 30625, Germany

**Keywords:** digitalization, door-to-needle time, ischaemic stroke, mobile application, process optimization, stroke

## Abstract

**Background:**

Timely treatment of ischaemic stroke with intravenous thrombolysis (IVT) and endovascular treatment (EVT) depends on efficient communication within a multiprofessional team. Mobile applications can streamline communication and documentation processes in acute stroke care.

**Objective:**

This study aims to implement a mobile app to facilitate digital documentation and communication in acute stroke care and evaluate its impact on documentation rates and treatment times. Furthermore, a user experience survey was performed to gather information about the app usage in an acute medical process.

**Design:**

The study is designed as an observational post-market clinical follow-up cohort study.

**Methods:**

The mobile app ‘Join’ was implemented in a tertiary stroke care centre. Feasibility was assessed by monitoring documentation rates and process times, that is, ‘door-to-needle time’ (DNT) and ‘door-to-groin time’ (DGT). All patients treated for suspected stroke or transitory ischaemic attack were included in a 6-month period prior to (T1) and a 3-month period after (T2) implementation of the Join app. User experience was evaluated through a standardized survey including technical features.

**Results:**

The interface between the mobile application, hospital information, picture archiving and communication, and quality assurance system as well as various magnetic resonance imaging/computer tomography scanners was implemented for acute stroke care. A total of 504 stroke patients was treated, 334 in T1 and 170 in T2. Of these, 65 received IVT and 87 received endovascular treatment (EVT). DNT (T1 vs T2, 27.5 vs 32 min, *p* = 0.987) and DGT (T1 vs T2, 50 vs 61.5 min, *p* = 0.481) were numerically longer during T2. Documentation rates increased threefold for all patients and 1.5 times for those receiving recanalization therapy. The survey revealed that documentation (76%) and case information retrieval (52%) were the most used app features, while other functionalities were less frequently utilized.

**Conclusion:**

Implementing a mobile app facilitated real-time digital documentation accessible to the entire stroke care team. The introduction of the app did not improve treatment times for patients receiving acute recanalizing therapies. We recommend systematic training programmes to promote user acceptance and effective use.

## Plain language summary

Improving stroke treatment efficiency with a mobile app: a pilot study

This study tested a mobile application designed to improve communication and documentation in acute stroke care. The application was implemented at a stroke care center to evaluate its impact on documentation completeness and treatment times. Results showed a significant increase in documentation rates but no effect on treatment times. The app’s most used features were documentation and retrieving patient information, while other functionalities were less popular. The study suggests that mobile apps can streamline acute stroke care and recommends training programs to ensure effective use by medical teams.

## Introduction

‘Time is brain’ and ‘Team is brain’ are the most important principles in acute ischaemic stroke care emphasizing the need to achieve recanalizing therapies, intravenous thrombolysis (IVT) and endovascular treatment (EVT), as fast as possible.^1,2^ The standard measures of these processes is the time interval between patients’ admission to the emergency room (ER) and the initiation of therapy as door-to-needle time (DNT), in case of IVT, and door-to-groin time (DGT), in case of EVT. An interdisciplinary team is necessary to conduct these therapies. IVT requires an intra-hospital team consisting of an ER physician, an ER nurse, a medical-technical radiology assistant, a neuroradiologist and a stroke unit (SU) nurse. It is crucial to streamline this process to ensure that the multiprofessional team acts in concert to optimize patients’ care. Moreover, documentation of this process is extremely important as well, in terms of quality assurance and for patients’ safety. The American Heart Association’s guidelines for treatment of early acute stroke recommend complete documentation, to be able to analyse treatment times effectively.^3^

Since the introduction of IVT and EVT, there have been numerous strategies for optimizing this workflow.^4–6^ Several of these (e.g. mobile thrombolysis kit) have already been implemented in our tertiary stroke care centre. This led to a significant reduction of DNT and DGT.^7,8^

Furthermore, digitalization is a promising game changer in emergency medical care. A lot of preliminary work has to be done to implement a digitalization tool in a highly sensitive acute medical process, for example equipping staff with smartphones, considering data protection regularities and coding of interfaces. The interfaces enable the data entered into the tool to be transferred to the hospital information system (HIS). Literature describes several approaches to digitalize acute stroke care processes and documentation.^9–13^ Most of these are smartphone applications, which have successfully reduced treatment times. However, these studies are only comparable to our centre to a limited extent, as they all started with a DNT above 50 min, whereas our median DNT is currently below 30 min.^7^

In this pilot study, we investigated the implementation of a mobile application at our tertiary stroke centre to evaluate its technical and procedural feasibility in a clinical use case. The primary objective was to implement the app technically, to optimize documentation rates and to evaluate the user experience and the impact on acute stroke treatment.

## Methods

The authors had full access to all study data and are responsible for data integrity and analysis. Underlying raw data are available upon reasonable request.

### Study population

**Figure 1.**
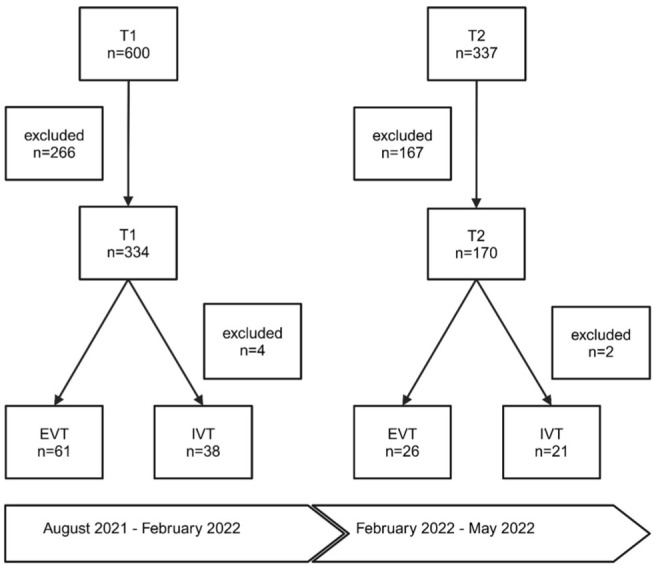
Overview of study workflow and population. In total, 937 patients have been collected of whom 433 had to be excluded from further analysis. In T1 and T2 patients, who had received an acute stroke therapy (IVT or EVT) in our centre, were analyzed in a subgroup. EVT, endovascular therapy; IVT, intravenous thrombolysis.

This study was conducted at the tertiary stroke care centre at Hannover Medical School, in two time intervals (T1 and T2) between August 19, 2021 and May 29, 2022 (Figure 1). T1 refers to the time before the app’s implementation between August 19, 2021 and February 15, 2022. T2 refers to the time after implementation between February 16, 2022 and May 29, 2022. In total, 937 consecutive patients with initially suspected stroke or transient ischaemic attack were admitted to the ER. Of these, 433 patients were excluded due to meeting the exclusion criteria shown in Table 1. To achieve a better comparability with previous process time analyses of our centre, two patients in the IVT group had to be excluded because they required intubation and mechanical ventilation before cerebral imaging and four due to secondary worsening of their neurological status after hospitalization.^7,8^ This resulted in a workflow analysis of 504 patients admitted with acute ischaemic stroke to our tertiary stroke care centre. In a 2:1 ratio, 334 patients were treated in T1, while 170 patients were treated in T2. In T1, 61 patients received EVT and 38 patients received IVT. In T2, 26 patients received EVT and 21 patients received IVT. In T1, workflow analysis was done retrospectively, whereas it was done prospectively in T2.

**Table 1.** Exclusion criteria and number of excluded patients in the total study group.

Exclusion criteria	Excluded patients (*n*)
Patient did not consent to scientific data analysis	207
Patient was not treated at SU or ICU (e.g. stroke mimics or subacute strokes)	65
In-house stroke	7
Intracranial haemorrhage	58
Retinal artery occlusion	64
Other diagnosis at admission	32

Exclusion criteria and number of excluded patients in the total study group.

ICU, intensive care unit; SU, stroke unit.

### Study procedures in T1

In T1, treatment of patients with a suspected ischaemic stroke was performed according to previously described standard operating procedures.^7,8^ To inform the interdisciplinary stroke team of an incoming stroke patient, the neurologist at the ER had to make a number of single phone calls. The documentation of process times and key parameters was paper based. This included for example: symptom onset, National Institute of Health Stroke Scale (NIHSS), admission time to ER, time of first cerebral imaging, start of IVT and the time of admission to a specialized ward (SU, Intensive Care Unit). For EVT, start of anaesthesia, time of groin puncture, time of first and last as well as number of recanalization attempts had to be documented.

The ER neurologists started the paper documentation with a document of up to 18 items. In case of an EVT the neuroradiology assistants completed the paper-based documentation. During this period, the necessary technical infrastructure for the app implementation was established according to the recently described technical workflow.^14^ Simultaneously, staff involved in acute stroke care were trained to use the app as a documentation and communication device. Furthermore, these professionals were equipped with smart phones.

### App facilities

The Join app (Allm Inc., Shibuya﻿, Tokyo, Japan) was implemented to streamline communication and documentation in acute stroke care. Detailed description of the technical preparations and integration process was described previously.^14^

For more convenient communication via the app, it was possible to perform video and phone calls, to chat in groups and to send crucial time stamps and parameters into a group chat of all members involved in acute stroke care. The time stamps and parameters were concordant with above mentioned, previous paper-based items.

Furthermore, the app features an image viewer providing the possibility to discuss acute stroke images with the consultant neurologist in charge locally independent.

### Study procedures in T2

T2 started directly after implementation of the app. The documentation had to be performed via app. It was recommended to document during the acute stroke care process, but it was also possible to do this afterward. The practical application includes a button in the HIS to transmit the case to the app where a form can be accessed to select the respective time stamp. The entry time is automatically recorded, but can also be set manually if entered later. The timestamps were also sent to the app’s group chat automatically, immediately informing the interdisciplinary team about the status of the patient. Furthermore, the documented time stamps were directly available in the HIS and integrated into the quality control registers.

Additionally, it was possible to use the group chat function, voice or video calls and the imaging viewer via the app to improve communication.

### User survey

A user survey with two focuses was performed. On the one hand, this survey included information about user behaviour regarding the app’s functions, for example how often it was used for documentation. On the other hand, the user experience was addressed with emphasis on the app’s technical suitability in this highly acute and time-dependent setting as a feedback for possible technical updates.

### Data acquisition

During the acute treatment of stroke patients, the ER neurologists documented key data on therapy procedures paper based (T1) or via the app (T2). Department of Neuroradiology assistants filled in the EVT data paper or app based. This was used to calculate the documentation rate. To analyse treatment times, missing time stamps were completed using patients’ admission record and anaesthesia protocol in case of an EVT. Additional data sources were clinical documentation records, the picture archiving and communication system (PACS) and emergency medical service (EMS) protocols for collection of the following clinical data: age, sex, NIHSS and modified Rankin Scale upon arrival, diagnosis at discharge, imaging modality, localization and aetiology of stroke and previous diseases, as well as cardiovascular risk factors. Furthermore, physicians and technical assistants of neurology, neuroradiology and anesthesiology documented factors of delay during acute stroke care. These factors of delay included: agitation and vomiting, hypertensive crisis, consultation of relatives, unknown onset, missing intravenous line (IVL), seizure, missing pre-notification by EMS, extended imaging, unclear indication, prolonged treatment in ER (>10 min) for not clearly documented reasons, waiting for imaging or report of imaging; and co-treatment by another department according to previous published factors of delay to achieve a better comparability.^7^

### Statistical analysis

Statistical analysis was performed using SPSS Statistics 27; IBM Corporation, Armonk, NY, USA. Descriptive statistics, including numbers and percentages, were utilized to characterize categorical variables. For non-normally distributed continuous variables, we reported the median and 25th–75th percentiles. Group comparisons were conducted using the Mann–Whitney *U* test for non-normally distributed continuous data and the Chi-square test for categorical data using 95% confidence intervals (CIs).

Results extracted from the user survey were presented descriptively using numbers and percentages. Figures were created using biorender.com.

## Results

### App implementation

**Figure 2.**
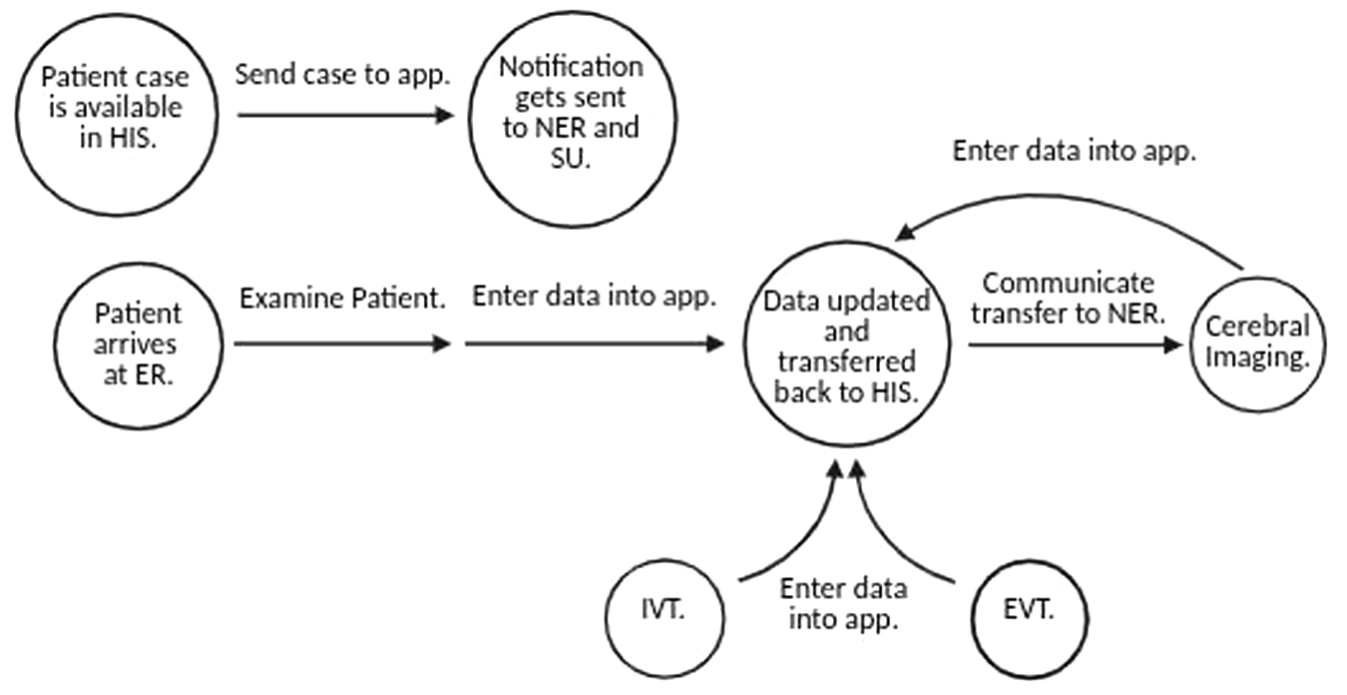
Overview of the technical workflow. Milestone events in the acute care of stroke patients are shown in circles and trigger the actions indicated by arrows. While the workflow describes the ER physician’s tasks, EVT data is provided by the Institute for Neuroradiology. ER, emergency room; EVT, endovascular therapy; HIS, hospital information system; IVT, intravenous thrombolysis; NER, Department of Neuroradiology; SU, stroke unit.

In a multidisciplinary team, consisting of computer scientists, neurologists and neuroradiologists, we successfully implemented a smart phone app in acute stroke care. Besides technical achievements, like coding interfaces to the HIS, PACS and the different imaging devices, we were able to find a way to digitalize one of the most time critical medical settings – acute stroke care (Figure 2).

### Documentation rate

**Figure 3.**
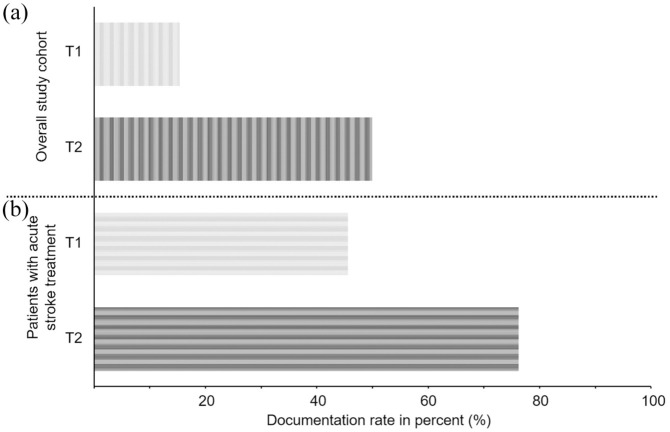
Documentation rates prior to and post-implementation of the app. (a) Documentation rate of the overall study cohort was higher in T2 than in T1. (b) Documentation rate of patients, who received an acute stroke treatment was higher in T2 than in T1. T1, time interval 1; T2, time interval 2.

In T1, the documentation of process times and key parameters had to be done paper based. In this interval, staff documented the necessary parameter of acute stroke treatment in only 51 of the 334 cases (15.3%). After the app implementation, the documentation rate increased significantly. In T2, 77 of 170 (45.6%; *p* < 0.001) cases were documented digitally (Figure 3(a)). Furthermore, for patients who received acute stroke therapy, the documented rate increased from 43 out of 86 (50%) to 32 out of 42 (76.2%, *p* = 0.005; Figure 3(b)).

### Study population and index stroke

Clinical characteristics including demographics and key data of index stroke are summarized in Table 2. We detected no relevant group differences. The prevalence of unknown time of onset was higher than expected, namely 54% in T1 and 51% in T2. This is due to the fact that all suspected stroke patients were considered, even if the stroke symptoms lasted longer than 24 h before admission. In T1, 17% and in T2 15% of the patients were secondary transferred to our centre to receive an EVT. A total of 90% of all patients in both groups received brain imaging at our site. The IVT rate at our site did not differ between the two groups. The proportion of IVT in the secondary transferred patients was not considered. Medical history and data on index stroke at discharge are presented in the Supplemental Material (Supplemental Table 1).

**Table 2.** Baseline characteristics in T1 and T2.

Characteristic	T1 (*n* = 334)	T2 (*n* = 170)
Female, *n* (%)	149 (45)	75 (44)
Age, median (25th–75th pct)	74 (62–82)	70 (59–81)
NIHSS, median (25th–75th pct)	2 (0–5)	2 (1–5)
Unknown time of onset, *n* (%)	181 (54)	86 (51)
Onset-to-door time, min (25th–75th pct)	127 (75–239)	102 (63–215)
Anterior circulation, *n* (%)	197 (71)	93 (67)
Admission by EMS, *n* (%)	273 (82)	138 (81)
Rate of secondary transferred patients, *n* (%)	55 (17)	25 (15)
Imaging on site, *n* (%)	299 (90)	135 (90)
Primary cCT, *n* (%)	136 (41)	57 (34)
IVT (in house), *n* (%)	42 (13)	23 (14)
EVT, *n* (%)	61 (18)	26 (15)
Diagnosis at discharge		
Stroke, *n* (%)	212 (64)	112 (66)
TIA, *n* (%)	77 (23)	32 (19)
Other (haemorrhagic strokes, stroke mimics, etc.), *n* (%)	45 (13)	26 (15)

Baseline characteristics in T1 and T2.

cCT, cranial computed tomography; EMS, emergency medical service; EVT, endovascular therapy; IVT, intravenous thrombolysis; NIHSS, National Institute of Health Stroke Scale; pct, percentiles; T1, time interval 1; T2, time interval 2; TIA, transitory ischaemic attack.

### Analysis of factors of delay in T1 and T2

To achieve better comparability of T1 and T2 stroke management processes, we examined factors of delay for the total study population as reported previously (Table 3).^7,8^ In T2, there were significantly more patients with a hypertensive crisis (T1 vs T2, 5.1% vs 10.6%, *p* = 0.02), missing IVL at arrival (T1 vs T2, 18% vs 24.7%, *p* = 0.049) and arrival without pre-notification by EMS (T1 vs T2, 18.3% vs 28.4%, *p* = 0.007). No other potential factors of delay showed any difference between the two time intervals. However, it should be noted that no factors of delay showed any difference between T1 and T2 when comparing the IVT- or EVT-subgroups (Supplemental Table 2).

**Table 3.** Comparison of factors of delay between T1 and T2.

4A: Factors of delay – total study cohort	T1 (*n* = 334)	T2 (*n* = 170)	*p*-Value
Missing pre-notification by EMS, *n* (%)	61 (18)	48 (28)	0.007
Missing IVL, *n* (%)	60 (18)	42 (25)	0.049
Unknown time of onset, *n* (%)	123 (37)	61 (36)	0.476
Consultation with relatives, *n* (%)	11 (3)	4 (2)	0.389
Co-treatment by another clinical department, *n* (%)	29 (9)	14 (8)	1.000
Waiting for brain imaging, *n* (%)	17 (5)	3 (2)	0.052
Extended imaging, *n* (%)	37 (11)	15 (9)	0.536
Hypertensive crisis, *n* (%)	17 (5)	18 (11)	0.020
Agitation or vomiting, *n* (%)	10 (3)	9 (5)	0.151
Epileptic seizure, *n* (%)	1 (0)	2 (1)	0.262
Unclear indication of acute therapy, *n* (%)	13 (4)	9 (5)	0.304

EMS, emergency medical service; IVL, intravenous line; T1, time interval 1; T2, time interval 2.

### Comparison of process times for IVT and EVT

**Figure 4.**
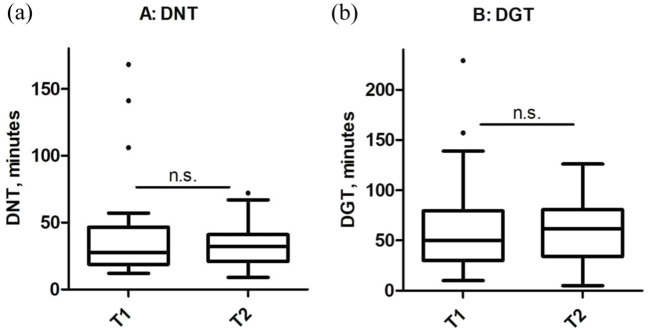
Comparison of time to treatment for recanalizing therapies in both cohorts (a) DNT or (b) DGT do not differ between both time intervals. DGT, door-to-groin time; DNT, door-to-needle time; T1, time interval 1 (August 2021–February 2022); T2, time interval 2 (February–May 2022).

In T1, 38 patients received an IVT and 61 patients an EVT. In T2, 21 patients were treated with IVT and 26 patients had an EVT. Median DNT was 28 min in T1 (25th–75th pct: 19–47 min) and 32 min in T2 (25th–75th pct: 21–41 min). DNT remained constant between T1 and T2 (median DNT T1: 28 min. 95% CI: 28–50 min vs median DNT. T2: 32 min 95% CI: 26–40 min; Figure 4(a)). The median DGT was 50 min (25th–75th pct: 30–80 min) in T1 and 62 min (25th–75th pct: 34–81 min) in T2. The median DGT value did not differ statistically significantly, but numerically and potentially with clinical relevance between T1 and T2 (T1: 50 min 95% CI: 50–70 min vs T2: 62 min 95% CI: 48–75 min; Figure 4(b)).

### User survey

Twenty-nine employees participated in the user-experience survey. Of these, 20 participants were from the Department of Neurology and 9 from the Institute for Diagnostic and Interventional Neuroradiology. The survey showed that the majority of the staff used the app for documentation (76%) and to get information about the cases (52%). In this first stage of implementation, staff did not use the voice or video call feature at all (Table 4). Following aspects were mentioned as the most frequent suggestions for improving the app: simplifying timestamp entry, improving chat clarity and a more intuitive user interface.

**Table 4.** User survey: 29 participants.

Tool	Usage, *n* (%)
Documentation	22 (76)
Case view	15 (52)
Messenger	9 (31)
Image viewer	4 (14)
Voice and video call	0 (0)
Proposals for optimization, *n* (%)	
Simplify timestamp entry	7 (24)
Improving chat clarity	5 (17)
More intuitive user interface	5 (17)
Automated case generation	1 (3)

## Discussion

In the rapidly evolving landscape of healthcare technology, the digitalization of critical medical processes has become increasingly important. This study focuses on the implementation of the App ‘Join’ in acute stroke care, a time critical medical process. In this study, we were able to digitalize the acute stroke care and documentation process, evaluate user experiences and assess the impact on crucial treatment timelines.

First of all, a major accomplishment of this digitalization effort was the implementation of a robust interface between the App ‘Join’ and the existing HIS, PACS, the quality assurance system and several magnetic resonance imaging and computer tomography scanners. This integration allows for seamless data flow and real-time synchronization of patient information, reducing data entry redundancy and improving the accuracy and consistency of patient records across systems. While not all tools, such as image viewer or video call, were frequently used, their availability demonstrates the app’s potential to address diverse needs within the stroke care process.

The transition from paper-based documentation in T1 to the app-based documentation led to a significant improvement in documentation rates. This increase was observed across the entire study cohort and for patients receiving acute stroke therapy. But to mention, after the app introduction the study team raised awareness about the documentation process, which will have contributed to higher documentation rates.

However, the advantages of the digitalization process extended beyond this fact. The real-time documentation ensured that patient information was up-to-date and readily available to all team members. Moreover, the automatic transmission of documented information to the HIS and quality assurance systems was a major benefit. Complete documentation is required to ensure compliance with guidelines and for quality improvement.^3^ Systematic reviews did not show any advantage in terms of time spent on documentation after implementation of electronic health records.^15^ However, we expect our system to do so in the long-term as it reduces ER physicians’ workload by widely automating documentation processes. This could facilitate the administrative tasks physicians have to spend so much time on.^16^ Lowering physicians’ workload has been proven to improve quality of care and patient safety.^17^ To sum up, digitalization of documentation streamlined the documentation processes and could have the potential to reduce duplicate data entry and minimize potential transcription errors.

Focusing on analysed factors of delay, in T2 we observed an increased occurrence of certain factors of delay in the overall study cohort, including missing pre-notification by EMS, missing IVL by admission and hypertensive crises. However, these factors did not differ significantly in the subgroup of patients who received acute recanalization therapies. Notably, while the stroke treatment times in T2 were not significantly longer, there was a trend towards prolongation. This outcome was contrary to our expectations, as the digitalization tool was anticipated to shorten treatment times. The analysis of delaying factors failed to provide a clear explanation for this trend. Testing the app prior to its implementation, we assessed the data entry to be quick and intuitive. In our opinion, this factor cannot account for an increase of several minutes in DNT and DGT. Furthermore, there may be other unmeasured factors contributing to the observed results. However, it is important to mention that our study has a relatively small sample size, which limits the ability to draw definitive conclusions. The inability of the digitalization tool to reduce stroke treatment times suggests that technological solutions alone may not be sufficient to optimize such a critical medical process. One potential approach to enhance the effectiveness of digitalization in acute stroke care is to combine it with systematic training programmes. Tahtali et al. introduced the guiding principle ‘team is brain’ and demonstrated the value of comprehensive stroke team training in improving treatment times and outcomes. A significant number of fatal errors are not caused by insufficient knowledge or technical ability, but from shortcomings in communication, interaction and decision-making. Therefore, a binding algorithm, Crew Resource Management (CRM) and regular simulation-based training are crucial to improving processes. CRM highlights the critical role of ‘non-technical skills,’ which it defines as cognitive, social and personal abilities that support and enhance technical expertise.^2^ This led to the suggestion that the implementation of new processes in acute stroke care, including digital solutions, should be carefully planned and supported by robust training initiatives.^18^

Following the successful technical implementation of the Join app and questionable results regarding its clinical benefits in our study, we conducted a user survey to obtain feedback on users’ experiences and to gain information on possible improvement strategies.^19^ This is very crucial due to the fact that user acceptance is very important for the effectiveness of digital systems.^20^ In our study, we explained the app’s individual functions to the users, but did not systematically train the users to use the app’s functions in acute stroke care. Our subsequent user survey revealed that many of the app’s features were either rarely or never used, whereas the documentation tool and the case report viewer have been the most commonly used features. Furthermore, the survey revealed that the team often suggested simplifying and clarifying the user surface. These results may suggest that a training, which includes the usage of the individual app functions within the acute care process, could potentially lead to more extensive use of the app’s various functions.

In literature, the Join app has already been used in a number of studies in acute stroke diagnosis and treatment with other use cases than in the current study. Some of these studies focused on the data image sharing feature of the app.^21–24^ Another study used the app in their ‘drip-and-ship’ workflow for inter-hospital communication, real-time tracking during patient transport and documentation of EVT milestones.^25^ Few studies have examined application scenarios similar to ours.^26–28^ These studies, while conducted in monocentric settings similar to ours, demonstrated significant reductions in stroke treatment times after the implementation of the Join app. However, a closer examination reveals markedly different baseline conditions, as these studies dealt with DNT exceeding 50 min before the app’s implementation, whereas our median DNT was 28 min in T1. This substantial disparity in initial treatment times underscores the limited comparability of these studies to our setting. Furthermore, this highlights the challenges in achieving further significant improvements when starting from an already optimized baseline. Nonetheless, our previous research gives us confidence that repetitive process analysis leads to further improvement.^7,8^ As the process is already highly condensed, it becomes more challenging in this context and the current form of the app may not be sufficient. Additional technical features, such as a single call activation – which the app is also capable of – may lead to further optimization. However, this has not yet been demonstrated and should be investigated in future studies.

## Limitations

Our study has several limitations that should be considered when interpreting the results. First of all, the higher awareness of the study team in T2 to use the app could have led to a higher documentation rate, potentially overestimating the impact of the digitalization tool as an observer bias. Future analyses conducted with a greater time distance from the implementation phase could provide more balanced insights. However, in T1 it was also repeatedly pointed out to carry out the documentation. Second, as a single-centre experience, our findings will not be generalizable to other healthcare settings with different resources and workflows. The workflow analysis was conducted in a 2:1 ratio, with T1 data obtained retrospectively and T2 data collected prospectively. This discrepancy in data collection methods introduces a potential bias, as retrospective data are generally considered less reliable than prospectively collected information. Furthermore, we had to exclude a large number of patients who did not agree to the general clinical data agreement used at our facility. This strongly affects the power of our analysis, due to reducing the sample size and introduces a potential selection bias by excluding more often patients with a more severe ischaemic stroke. Finally, the subgroups of patients treated with IVT or EVT were rather small, resulting in insufficient statistical power to draw definitive conclusions about the impact of the digitalization tool on these specific interventions.

## Conclusion

In conclusion, our study represents the first implementation of the Join app for acute stroke care in Germany, successfully integrating the application with all necessary interfaces into our HIS. Our user survey revealed that the app’s full range of functions was not used. After the introduction of the app, the treatment times for patients receiving acute recanalizing therapies showed a trend towards prolongation. This does not meet our expectation, because the usage of the app only requires a few clicks and does not affect the workflow in this manner. These findings lead to the suggestion that digital solutions alone may not be sufficient to improve a complex medical setting. We propose that systematic training programmes, such as a stroke team training, are crucial to accompany the digitalization process, potentially enhancing the adoption and effective use of such tools. This approach could bridge the gap between technological implementation and practical application, highlighting the complex interplay of various elements in acute stroke management and the need for a holistic approach to process optimization beyond digital solutions alone.

## Supplemental Material

sj-docx-1-tan-10.1177_17562864251379215 – Supplemental material for Workflow optimization in acute stroke therapy using a mobile application – a pilot study Supplemental material, sj-docx-1-tan-10.1177_17562864251379215 for Workflow optimization in acute stroke therapy using a mobile application – a pilot study by Frieso Geerd Stevens, Hans Worthmann, Clara Zoe Fricke, Mareike Schulze, Gerrit M. Grosse, Maria M. Gabriel, Jan Beneke, Sybille Schiele, Ramona Schuppner, Karin Weissenborn, Friedrich Götz, Anna-Lena Boeck and Johanna Ernst in Therapeutic Advances in Neurological Disorders

sj-docx-2-tan-10.1177_17562864251379215 – Supplemental material for Workflow optimization in acute stroke therapy using a mobile application – a pilot study Supplemental material, sj-docx-2-tan-10.1177_17562864251379215 for Workflow optimization in acute stroke therapy using a mobile application – a pilot study by Frieso Geerd Stevens, Hans Worthmann, Clara Zoe Fricke, Mareike Schulze, Gerrit M. Grosse, Maria M. Gabriel, Jan Beneke, Sybille Schiele, Ramona Schuppner, Karin Weissenborn, Friedrich Götz, Anna-Lena Boeck and Johanna Ernst in Therapeutic Advances in Neurological Disorders

## Appendix

### Abbreviations

cCT cranial computed tomography

CRM Crew Resource Management

DGT door-to-groin time

DNT door-to-needle time

EMS emergency medical service

ER emergency room

EVT endovascular treatment

HIS hospital information system

IVL intravenous line

IVT intravenous thrombolysis

NIHSS National Institutes of Health Stroke Scale

PACS picture archiving and communication system

SU stroke unit

T1 time interval 1 (August 2021–February 2022)

T2 time interval 2 (February–May 2022)

TIA transitory ischemic attack

## Declarations

**Ethics approval and consent to participate:** The Ethics Committee of Hanover Medical School approved this study in accordance with the principles of the Declaration of Helsinki and data protection law of Lower Saxony §13 (No. 9869_BO_SK_2021) on August 5, 2021. The Ethics Committee waived the requirement for a particular written informed consent if patients agreed to scientific data analysis in the general hospital service agreement.

**Consent for publication:** Not applicable.

**Availability of data and materials:** On reasonable request.
