# Supplementary material for: Workflow optimization in acute stroke therapy using a mobile application – a pilot study
Source: Ther Adv Neurol Disord. 2025 Oct 18;18:17562864251379215. doi: 10.1177/17562864251379215 (PMC12541197; doi:10.1177/17562864251379215)
Supplement: sj-docx-2-tan-10.1177_17562864251379215 – Supplemental material for Workflow optimization in acute stroke therapy using a mobile application – a pilot study [file sj-docx-2-tan-10.1177_17562864251379215.docx]

Supplement Table 1: Medical history and data on index stroke at discharge

| Medical History | T1 (n=334) | T2 (n=170) | p |
| --- | --- | --- | --- |
| Arterial hypertension, n (%) | 249 (75) | 122 (72) | 0.468 |
| Diabetes mellitus, n (%) | 73 (22) | 34 (20) | 0.618 |
| Atrial fibrillation, n (%) | 100 (30) | 38 (22) | 0.068 |
| Chronic heart failure, n (%) | 51 (15) | 23 (14) | 0.593 |
| Hyperlipidemia, n (%) | 242 (73) | 113 (67) | 0.149 |
| History of stroke or TIA, n (%) | 86 (26) | 38 (22) | 0.393 |
| Myocardial infarction, n (%) | 24 (7) | 7 (4) | 0.173 |
| Nicotine abuse, n (%) | 59 (17) | 31 (18) | 0.886 |
| Stroke etiology according to TOAST criteria (without stroke mimics) | T1 (n=289) | T2 (n=144) |  |
| Large-artery atherosclerosis, n (%) | 35 (12) | 17 (12) |  |
| Cardioembolism, n (%) | 28 (10) | 21 (15) |  |
| Small-vessel occlusion, n (%) | 86 (30) | 33 (23) |  |
| Other etiology, n (%) | 11 (4) | 4 (3) |  |
| Unclear etiology, n (%) | 129 (45) | 69 (48) |  |

Supplement Table 2: Factors of delay in the IVT- and EVT-subgroup

| Factors of delay | IVT  T1 (n=38) | IVT  T2 (n=21) | EVT  T1 (n=61) | EVT  T2 (n=26) |
| --- | --- | --- | --- | --- |
| Missing pre-notification by EMS, n (%) | 4 (11) | 3 (14) | 3 (5) | 0 (0) |
| Missing IVL, n (%) | 4 (11) | 2 (10) | 2 (3) | 0 (0) |
| Unknown time of onset, n (%) | 15 (40) | 4 (19) | 27 (44) | 10 (39) |
| Consultation of relatives, n (%) | 5 (13) | 2 (10) | 4 (7) | 2 (8) |
| Co-treatment of other department, n (%) | 1 (3) | 0 (0) | 2 (3) | 1 (4) |
| Waiting for brain imaging, n (%) | 6 (16) | 1 (5) | 3 (5) | 0 (0) |
| Extended imaging, n (%) | 5 (13) | 2 (10) | 14 (23) | 4 (15) |
| Hypertensive crisis, n (%) | 11 (29) | 7 (33) | 2 (3) | 4 (15) |
| Agitation or vomiting, n (%) | 4 (11) | 3 (14) | 2 (3) | 3 (12) |
| Epileptic seizure, n (%) | 1 (3) | 0 (0) | 0 (0) | 1 (4) |
| Unclear indication of acute therapy, n (%) | 7 (18) | 3 (14) | 5 (8) | 2 (8) |

Abbreviations: T1 = time interval 1; T2 = time interval 2; NER = Department of neuroradiology; IVL = intravenous line, EMS = emergency medical service; ER = emergency room
